# Development and Evidence of the Validity of the Condom Use Attitudes Scale for Youth and Adults in a Chilean Context

**DOI:** 10.3389/fpsyg.2021.727499

**Published:** 2021-12-01

**Authors:** Rodrigo Ferrer-Urbina, Patricio Mena-Chamorro, Geraldy Sepúlveda-Páez, Marcos Carmona-Halty

**Affiliations:** Escuela de Psicología y Filosofía, Universidad de Tarapacá, Arica, Chile

**Keywords:** attitudes toward condom use, HIV/AIDS, sexual risk behavior, ESEM, Psychometric scales development

## Abstract

Condom use is the most effective preventive behavior against HIV transmission, and its inadequate use is a public health problem that occurs mostly among youth and young adults. Although there are scales that measure condom use, those that exist correspond to English-speaking developments or do not have psychometric evidence to support them, so it is possible that the available adaptations of instruments do not adequately reflect the phenomenon in the Chilean population. Thus, the study aims to develop a scale to assess attitudes toward condom use in Chilean youth and young adults. Initially, a sample of students between 18 and 39 years (*n* = 520) was used for debugging the instrument. Then, a second sample was taken from the general population aged 18 to 40 (*n* = 992) to confirm the factor structure of the proposed model. The final scale has 10 items and 3 attitudinal dimensions (affective, cognitive, and behavioral). The results show that the identified structure provides adequate levels (ω > 0.7) or at least sufficient of reliability (ω > 0.6) and presents evidence of validity, based on the internal structure of the test, through ESEM (CFI = 0.993; TLI = 0.984; RMSEA = 0.056). In addition, evidence of validity was obtained based on the relationship with other variables and strong invariance between the scores of men and women. It is concluded that the scale developed has adequate psychometric properties to assess, in brief form, condom use attitudes in equal samples for research and screening purposes.

## Introduction

Human immunodeficiency virus and acquired immunodeficiency syndrome (hereafter HIV/AIDS) is a problem that affects millions of people, mainly in low- and middle-income countries (World Health Organization, 2016). Notwithstanding global efforts to prevent its transmission, the number of carriers continues to increase, with 120,000 new cases registered during 2019 in Latin America alone (Joint United Nations Program on HIV/AIDS, 2019; Pan American Health Organization, 2019).

In Chile, the context is similar, showing a systematic increase in the number of HIV/AIDS reports, with the highest prevalence figures in the regions of Arica and Parinacota, Metropolitan and Tarapacá (Cáceres-Burton, 2019). The main affected people are between 20 and 29 years, being 40.4% of the total number of new cases confirmed during 2017 (Instituto de Salud Pública, 2016; Barrera-Herrera and Vinet, 2017; Cáceres and Pino, 2018; Cáceres-Burton, 2019).

This scenario has prompted multiple governmental efforts to reduce sexual risk behaviors (hereafter, SRB) (i.e., sex with inadequate condom use, sex under the influence of alcohol and drug use, and multiple sexual partners), because they are the main way of transmission (Kilwein and Looby, 2018; Yi et al., 2018). Despite these efforts, the results of prevention interventions and programs remain insufficient, especially in the heterosexual population (Kilwein et al., 2017; Habel et al., 2018).

Among the HIV/AIDS prevention’s mechanisms, the one with most support and consensus is the use of condoms (Johnson et al., 2018), since they provide an impermeable barrier for sperm-sized particles and HIV and STI pathogens, making their systematic and correct use effective for prevention (Smith et al., 2015). However, the literature points out that a high proportion of young people report not using condoms consistently, which increases the risk of HIV/STI infection (Pinyaphong et al., 2018). For example, in the Chilean context, only 22.1% of young people between 20 and 24 years and 11.5% of adults between 25 and 29 years report always using female or male condoms during the last 12 months (Ministerio de Salud, 2017).

Evidence shown that there are multiple psychosocial factors that directly or indirectly influence potential condom use, including which sexual communication (Javier et al., 2018), perceived risk (Elshiekh et al., 2020), type of sexual partner (formal or informal) (Bryan et al., 2017), previous condom use, knowledge, self-efficacy, and attitude toward condom use (Sheeran et al., 2016; Janulis et al., 2017; Teye-Kwadjo et al., 2017). While all these characteristics impact condom use, the factor that has evidenced the most significant impact on condom use is attitude (Sheeran et al., 1999; Mbelle et al., 2018).

Favorable attitudes toward condoms have been shown to lead to healthy sexual behaviors (De Torres, 2020; Kim et al., 2021), while unfavorable attitudes decrease the likelihood of condom use (Ajayi et al., 2019; Elshiekh et al., 2020) and are associated with other sexual risk behaviors, such as multiple sexual partners (Shamu et al., 2020) and sex under the influence of alcohol or drugs (Davis et al., 2020).

Although there are several instruments to measure attitudes toward condom use (e.g., Brown, 1984; Sacco et al., 1991; Helweg-Larsen and Collins, 1994; Hanna, 1999; Neilands and Choi, 2002; Crosby et al., 2010; Hollub et al., 2011), and some of these have been adapted for Spanish-speaking populations (Vallejo-Medina et al., 2019; Plaza-Vidal et al., 2020), they have some characteristics (e.g., scales specific to a social group; scales specific to a female or male condom; scales developed in a foreign language) (Beachy et al., 2020) which reduce their general usability and threaten the validity of their interpretations in particular settings.

Therefore, evidence shown that cultural differences can affect people’s perceptions and attitudes toward condoms (Mileti et al., 2018; Patterson, 2019), and there are no instruments with evidence of validity to support their uses and interpretations in chilean population, according to current standards (American Educational Research Association, American Psychological Association, and National Council on Measurement in Education, 2014). This study aims to develop a scale to measure the attitude toward condom use, with evidence of validity for its use in Chilean youth and young adults.

Considering that multiple attitudinal assessment models have been proposed in the literature, such as the theory of reasoned action (TRA; Fishbein and Ajzen, 1975) and planned action (TPB; Ajzen and Madden, 1986) models, given that we intend to develop a brief scale focused on past behavior, we opted to use a simpler attitudinal model, specifically the 3-component model: (1) Cognitive component: thoughts and beliefs held about the object; (2) Affective, referring to the affective/emotional evaluation held about the object; (3) and Behavioral, referring to the way of acting that allows making evaluations about the attitudinal object (Zanna and Rempel, 1988). Furthermore, given that some authors have argued that attitude can be analyze as a global construct (e.g., La Trobe and Acott, 2000; Gaborieau and Pronello, 2021), it was decided to contrast this possibility as an alternative one-dimensional model, to test the support to a general factor.

## Materials and Methods

An instrumental study with a cross-sectional design was conducted (Ato et al., 2013).

### Participants

Two samples were used for this study: (a) one of university students between 18 and 39 years, and (b) one of the general population between 18 and 40 years. The participants of both samples were chosen using non-probability sampling strategies (Otzen and Manterola, 2017). Sample (a), collected using a time-space strategy, is composed of 520 young adults (*M* = 22.7; *SD* = 3.5) from the city of Arica, 264 (50.7%) were female and 253 (48.6%) were male, where 84.2% (*n* = 438) reported being heterosexual and 61.3% (*n* = 320) reported having used protective barrier methods in the last 2 years; sample (b), collected using a quota strategy (i.e. sex, educational level and city), according to the baseline proportions provided by the 2017 CENSUS results (Instituto Nacional de Estadística, 2018), is composed of 992 young adults (*M* = 23.3; *SD* = 4.6), 514 (52.0%) females and 464 (46.7%) males, from the cities of Arica (22%; *n* = 218), Iquique (14.3%; *n* = 142), Alto Hospicio (9.5%; *n* = 94), Antofagasta (37.1%; *n* = 368) and Calama (17.1%; *n* = 170). A total of 82.4% (*n* = 818) said they were heterosexual, and 56.8% (*n* = 565) said they had used protective barrier methods in the last 2 years. Sociodemographic details are shown in Supplementary Table 1.

### Instruments

Condom Use Attitude Scale (CUAS): developed ad-hoc to evaluate the subjective valence of prevention behaviors and use of protective barriers through three attitudinal dimensions: (a) affective, (b) behavioral, and (c) cognitive. The response options are in a Likert format of 4 ordered categories (1 = “Strongly disagree” to 4 = “Strongly agree”). The statements refer to negative attitudes/behavior toward condom use. Therefore, high scores suggest an unfavorable attitude toward condom use.

Initially, 60 items (20 for each dimension) were outlined and assessed by three expert judges (two health professional judges and one judge with psychometric experience) based on grammatical adequacy (coherence and clarity) and construct representativeness. Judges individually scored “1, 0, or –1,” where “1” represents grammatical adequacy and construct representativeness of the item. After that, means were calculated, and items with means less than or equal to 0 were eliminated. A pilot study was applied online (*n* = 110), with a 32-item version, from which those items with values below 0.30 in the corrected homogeneity coefficient were iteratively eliminated. Finally, a 17-item version was obtained, applied in samples (a) and (b) for this study. The final version (see Supplementary Protocols 1, 2) and its psychometric evidence are reported in the results section.

Sexual risk behaviors scale (Ferrer-Urbina et al., 2018): is a 12-item instrument designed to assess 3 dimensions of sexual risk behaviors: (a) sexual activity with multiple partners (4 items), (b) inappropriate or insufficient use of protective barriers (4 items), and (c) sexual activity under the influence of alcohol and drugs (4 items). The response options have a Likert format of four ordered categories (1 = “Strongly Disagree” to 4 = “Strongly Agree”) to avoid acquiescence and to force decision making given their attitudinal character. Response options are conditional on reporting only behaviors in the past 2 years. The scale stated evidence of validity based on internal structure and adequate levels of reliability (ω > 0.8) (Ferrer-Urbina et al., 2018).

### Procedure

Eight fifth-year psychology students were trained to apply the questionnaires in pencil and paper format to collect the samples (a). Participants were contacted between March and May 2018 in recreational areas of higher education institutions (e.g., reading areas, interior courtyards, library, and others.). The response procedure lasted approximately 15 min.

Twenty surveyors were trained and assigned in the study cities to collect the sample (b). Participants were contacted between March and July 2019 in the busiest areas of each city. The questionnaires were self-administered in pencil and paper format. The answer procedure lasted 15–20 min.

In both sample collection processes, the questionnaire was provided with an informed consent, where the research objectives, the rights of the participants, anonymity, and confidentiality of their participation were established. Volunteers responded on the spot without any reward or incentive. Anonymity was guaranteed by the return of the questionnaire in a sealed envelope, without any personal identification. The research was approved by the Scientific Ethics Committee of the Universidad de Tarapacá, within the framework of ANID’s grant (Fondecyt de iniciación 11170395).

### Data Analysis

Initially, to establish the empirical dimensionality of the test with sample (a), a parallel analysis was realized, based on exploratory factor analysis (EFA) with minimum residual estimation method, 20 replicates, and based on average eigenvalues of the simulated data. Then, to establish the preliminary evidence of validity based on the internal structure of the test, an ESEM with GEOMIN rotation (Asparouhouv and Muthén, 2009) and WLSMV estimation method, which is robust with non-normal discrete variables (DiStefano and Morgan, 2014; Li, 2016), was performed from the polychoric correlation matrix, given the ordinal structure of the data (Barendse et al., 2015). Subsequently, in order to obtain a shorter and optimized scale, it was iteratively debugged by eliminating items based on three criteria: (1) retention of items with strong factor loadings (λ > 0.5); (2) elimination of redundant items (Abad et al., 2011) and (3) elimination of items with strong cross-loadings (>0.3) (Muthén and Asparouhov, 2012; Xiao et al., 2019).

To test the empirical dimensionality of the debugged version, establish the evidence of the internal structure, contrast alternative models, assess the stability of the scale, and obtain evidence of validity based on the relationship with other variables, sample (b) was used. The dimensionality of the debugged version was tested with parallel analysis. The debugged factor structure was tested with ESEM with GEOMIN rotation and an alternative one-dimensional model, both with the WLSMV estimation method and based on polychoric correlations. Reliability was also estimated for each dimension and with Cronbach’s alpha and McDonald’s omega coefficients, both in non-ordinal versions (Viladrich et al., 2017). Measurement invariance between persons of different sexes was assessed using a multi-group ESEM (i.e., metric and scalar). Increases in RMSEA below 0.010 were considered evidence of invariance (Chen, 2007). Finally, evidence of validity based on the relationship with other variables was established, using SET-ESEM (with GEOMIN rotation, WLSMV estimator and from polychoric correlations), between the dimensions of the CUAS, the dimensions of the sexual risk behavior scale (Ferrer-Urbina et al., 2018), and a single-item scale on condom use in the past 2 years.

The overall organization of the models was assessed following the cut-point recommendations stated by Schreiber (2017) for the comparative fit index (CFI), Tucker-Lewis index (TLI), and root mean squared error of approximation (RMSEA) (e.g., CFI > 0.95; TLI > 0.95; RMSEA < 0.06).

Reliability and parallel analysis were performed with Jamovi version 2.0.0 (The Jamovi Project, 2020) and ESEM, SET-ESEM and Measurement invariance were performed with Mplus version 8.2 (Muthén and Muthén, 1998-2017).

Sample (a) showed 0.017% of missing data, while the sample (b) showed 0.027% of missing data. The Pairwise method was used for the missing data handling.

## Results

### Exploratory Analysis With Sample (a)

Initially, parallel analysis suggested four factors with eigenvalues over the extraction of random variables, but one of them was explained only by two items. Then, excluding those two items, a second parallel analysis suggest three factors (see Supplementary Figure 1), showed good statistic’s fit ($\chi_{D\,F=63}^{2}=161.437;$TLI = 0.929; RMSEA = 0.056) and factorials loadings that fit with the proposed structure (see Supplementary Table 2). Then, two ESEM models were estimated, one with the original version (M1) (three dimensions and 15 items) and the other with the debugged version (M2a) (three dimensions and 10 items), using the reported criteria in data analysis section. The original model (M1) showed good statistic’s fit, according to the standards recommended in the literature ($\chi_{D\,F=63}^{2}=179.230;$CFI = 0.982; TLI = 0.969; RMSEA = 0.060; SRMR = 0.031) (Schreiber, 2017). However, M2a ($\chi_{D\,F=18}^{2}=61.566;$CFI = 0.992; TLI = 0.979; RMSEA = 0.068; SRMR = 0.023) shown a better structure‘s fit, except for the RMSEA index (see Supplementary Table 3). The M2a model also showed good factor loadings on each factor (affective, λ = 0.55–0.91; behavioral, λ = 0.58–0.83; cognitive, λ = 0.47–0.79) and low levels of cross-loadings (affective, λ = −0.07–0.11; behavioral, λ = −0.04–0.22; cognitive, λ = −0.07–0.04). Structural relationships between dimensions were moderate (*r* > 0.30) and mild (*r* > 0.10) (Cohen, 1988; see Supplementary Table 4).

### Dimensionality and Evidence of Validity Based on Internal Structure

In sample (b), from the debugged (10 items version), a parallel analysis, an ESEM (M2b) (three dimensions with 10 items) and one-dimensional (M3) models was tested. The parallel analysis suggested three factors with eigenvalues over the extraction of random variables (see Supplementary Figure 2). Only M2b ($\chi_{D\,F=18}^{2}=71.996;$CFI = 0.994; TLI = 0.984; RMSEA = 0.055; SRMR = 0.016) showed good fit. M3 ($\chi_{D\,F=40}^{2}=1480.23;$CFI = 0.829; TLI = 0.780; RMSEA = 0.205; SRMR = 0.115) showed fit indicators far away from the standards recommended in the literature. Details of the validity based on the internal structure of the test are shown in Supplementary Table 3.

### Standardized Factor Loadings, Factorial Covariations and Score’s Reliability

Table 1 presents the factor loadings with their corresponding factorial covariates and reliability coefficients of the three-dimensional covariate (M2b) and the one-dimensional (M3) models in the sample (b).

**TABLE 1:** Descriptive information of the CUAS and factor loadings resulting from ESEM and CFA in general population sample.

Attitude toward condoms	Mean (*SD*)	*S*	K	Factor loadings				Reliability	
				M2b			M3	α if item is dropped	ω if item is dropped
				A	B	C	–		
**Affective (A)**									
**Es difícil disfrutar del sexo cuando se usa preservativo.** (It’s hard to enjoy sex when you use a condom).	2.44 (0.98)	–0.57	6.55	0.830**	0.080	–0.018	0.819**	0.731	0.731
**Siento que el preservativo disminuye mi satisfacción sexual.** (I feel that the condom decreases my sexual satisfaction).	2.51 (0.97)	–2.28	6.22	0.924**	0.011	–0.010	0.854**	0.681	0.681
**Las personas obtienen más placer en las relaciones sexuales sin preservativo.** (People get more pleasure from sex without a condom).	2.63 (1.06)	–3.06	–7.54	0.608**	–0.014	0.271**	0.679**	0.849	0.848
**Behavioral (B)**									
**Evito usar preservativo cada vez que me lo permiten.** (I avoid using a condom every time I’m allowed).	2.17 (1.03)	4.60	–6.82	0.356**	0.498**	0.005	0.622**	0.504	0.504
**No suelo llevar preservativos cuando tengo un encuentro sexual.** (I don’t usually wear a condom when I have a sexual encounter).	2.26 (1.09)	3.40	–8.03	–0.012	0.776**	–0.017	0.494**	0.503	0.502
**Tendría relaciones sexuales aun cuando mi pareja se negará a usar preservativo.** (I would have sex even if my partner refused to use a condom).	2.33 (1.05)	1.24	–783	0.174*	0.381**	0.183**	0.525**	0.588	0.588
**Cognitive (C)**									
**Creo que el preservativo debieran usarlo solo las personas promiscuas.** (I think the condom should only be used by promiscuous people).	1.63 (0.93)	17.54	5.38	–0.004	0.113	0.714**	0.670**	0.725	0.729
**El uso de preservativos es solo para relaciones pasajeras.** (The use of condoms is only for temporary relations).	1.84 (0.99)	11.05	–3.08	–0.003	0.084	0.775**	0.707**	0.690	0.695
**Pienso que el preservativo es innecesario en las personas sanas.** (I think condoms are unnecessary in healthy people).	1.65 (0.89)	15.53	3.34	–0.005	–0.003	0.838**	0.705**	0.687	0.699
**Creo que sugerir el uso del preservativo genera desconfianza.** (I think that suggesting condom use creates distrust).	1.69 (0.90)	14.58	1.87	0.079	–0.009	0.673**	0.598**	0.754	0.756

				Correlations			–	α index	ω index

**Affective (A)**	2.53 (0.86)	–2.26	–5.79	–			–	0.822	0.826
**Behavioral (B)**	2.25 (0.80)	0.32	–3.79	0.445**	–		–	0.631	0.635
**Cognitive (C)**	1.69 (0.71)	1.16	1.34	0.318**	0.459**	–	–	0.770	0.774

SD, Standard Deviation; S, Skewness; K, Kurtosis. **p < 0.001; M2b, ESEM with three covariate factors; 10 items, M3; One-dimensional CFA, 10 items.

*Bold values indicate the expected dimension of the factor loadings.*

*p < 0.05.

M2b model, factor loadings were shown to be adequate or at least sufficient representations of each factor (affective, λ = 0.60–0.92; behavioral, λ = 0.38–0.77; cognitive, λ = 0.67–0.83) and to have low levels of cross-loadings (affective, λ = –0.01–0.35; behavioral, λ = –0.01–0.11; cognitive, λ = –0.01–0.27). Structural relationships between dimensions were moderate (*r* > 0.30) (Cohen, 1988). Reliability estimates were adequate or at least sufficient (ω > 0.70; α > 0.70) (Cho and Kim, 2015), although slightly lower (ω > 0.60; α > 0.60), in the case of the behavioral dimension.

In M3 model, factor loadings were shown to be adequate representations of one-dimensional factor (λ = 0.49 –0.85). Details of standardized factor loadings, factorial covariations and score’s reliability are shown in Table 1.

### Factorial Invariance and Evidence of Validity Based on the Relationship With Other Variables

The three-dimensional covariate model (M2b) was used to estimate tests of invariance between men and women, with sample (b). The metric (RMSEA = 0.049) and scalar model (RMSEA = 0.054) (restricted) compared to the configural model (RMSE = 0.049) (unrestricted) showed no relevant changes in the RMSEA differential, with the equivalence between factor loadings and factor thresholds being sustainable for both groups. Details of factorial invariance test are shown in Supplementary Table 3.

**FIGURE 1:**
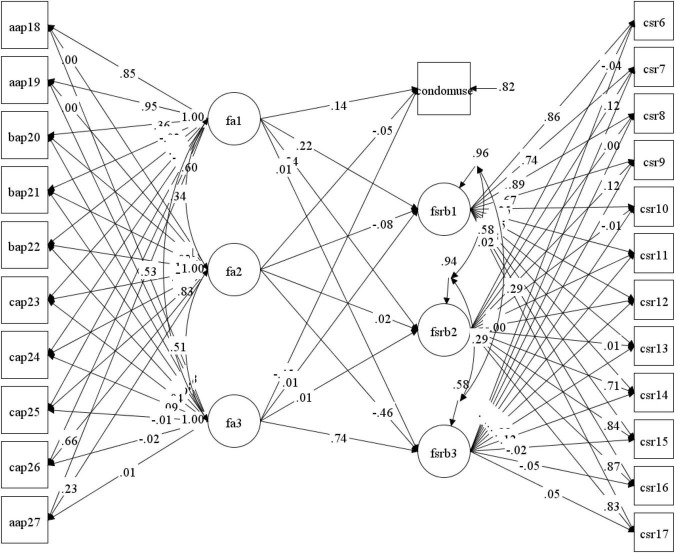
Graphical representation of the SET-SEM model; Fa1, Affective; Fa2, Cognitive; Fa3, Behavioral; Fsrb1, Sexual activity with multiple partners; Fsrb2, Sexual activity under the influence of alcohol or drugs; Fsrb3, Inappropriate use of protective barriers; condomuse, Condom use.

Finally, the SET-ESEM model that estimated the association between the latent dimensions of the attitudes regarding condom use scale and the sexual risk behaviors scale, in addition to the observed variable condom use (see Figure 1), showed satisfactory comparative and absolute fit indexes ($\chi_{D\,F=181}^{2}$ = 395.128; CFI = 0.986; TLI = 0.980; RMSEA = 0.035, [CI = 0.030–0.039]). According to observed relationships, the affective component had mild direct effects on sexual activity with multiple partners (γ = 0.216, *p* < 0.001); sexual activity under the influence of alcohol or drugs (γ = 0.237, *p* < 0.001); and a slight effect on condom use (γ = 0.141, *p* = 0.012). The behavioral component had a large effect on inappropriate use of protective barriers (γ = 0.736, *p* < 0.001) and a moderate inverse effect on condom use (γ = –0.452, *p* < 0.001). Finally, the cognitive component had a moderate inverse effect on inappropriate use of protective barriers (γ = –0.455, *p* < 0.001). Details of standardized relationships between latent dimensions are shown in Supplementary Table 5.

## Discussion

The purpose of this study was to develop a scale to measure attitudes toward condom use, with evidence of validity for its use in youth and young adults in a Chilean context. The fit statistics of the covariate model (M2), both for the sample of students (a) and the general population (b), the size of the factor loadings, and the absence of relevant cross-loadings (except for one item of the behavioral dimension that showed a slightly higher cross-loading with the affective dimension), support the multidimensional structure of the model (three dimensions) and provide evidence of validity based on the internal structure for the adequate interpretation of the scores. The one-dimensional model (M3) cannot be used as a plausible explanation of the instrument so that the scores could be interpreted from its specific aspects and do not from the combination of its dimensions. The estimates of the reliability coefficients allow us to argue that each dimension has an adequate or satisfactory level of consistency, except for the behavioral dimension, which has a slightly lower estimate, which is to be expected given the small number of items and the effect this has on reliability estimates based on internal consistency.

According to the invariance standards suggested by Chen (2007), it is possible to sustain metric and scalar invariance of the measures according to the gender of the participants (i.e., strong invariance). Therefore, it is possible to apply the scale to men and women and compare them since the factor loadings were equivalent between the two groups and the dimensions had the same differential variability between the sexes. Although it is crucial to consider the effects produced by socio-structural variables such as gender roles on condom use (Casique, 2019), this scale allows comparisons between men and women with an adequate interpretation of the results obtained.

In terms of evidence of validity based on association with other variables, the dimensions of the CUAS were observed to explain sexual risk behaviors and condom use partially, as had been noted in previous research (Smith et al., 2015; Johnson et al., 2018; Kilwein and Looby, 2018; Mbelle et al., 2018; Yi et al., 2018; Ajayi et al., 2019). The observed relationships were in the expected direction, except for the relationship between the affective component with condom use. This exception could be attributed to the involvement of other variables of relevance to condom use (i.e., risk perception, knowledge of risky sexual behaviors, communication with sexual partners) that overlap with people’s affective attitudes toward condom use. Therefore, it is possible that people who manifest a negative attitude might not necessarily reduce condom use, especially if they believe they are susceptible to the risk of transmission or are aware of the risks associated with non-use (Sheeran et al., 2016; Janulis et al., 2017; Teye-Kwadjo et al., 2017; Elshiekh et al., 2020).

The main constraint of this study corresponds to the size and representativeness of the sample. Although two samples were used, both were non-probabilistic. Therefore, there is no guarantee of the adequacy of the generalization to population values. It is suggested that further psychometric studies be conducted using this instrument in other populations (e.g., high-risk populations, new countries, and migrant populations) and medical, health, and educational contexts. Finally, we need to be careful about the relation of the self-report item of condom use, since the extremely large effect size can be explained, in part, by the fact that the behavioral dimension of the scale makes direct references to condom use behavior.

The inclusion of this scale in an evaluation protocol in health services or educational establishments could be helpful since the information provided by this measurement instrument will make it possible to identify groups of subjects that require specific preventive interventions. Consequently, current interventions and strategies that promote sexual health among youth and adults could be complemented and improved.

The final version (10 items) of the scale of attitudes toward condom use presents evidence of reliability and validity (i.e., based on the internal structure of the test and the association with other variables). The initial evidence suggests that the current scale constitutes a new brief instrument developed with contemporary psychometric techniques and establishes an updated and alternative proposal to assess attitudes toward condom use, which can also be used to develop studies on the psychological factors involved in sexual behaviors.

## Data Availability Statement

The original contributions presented in the study are included in the article/Supplementary Material, further inquiries can be directed to the corresponding author/s.

## Ethics Statement

The studies involving human participants were reviewed and approved by the Research Ethics Committee of the Universidad de Tarapacá. The patients/participants provided their written informed consent to participate in this study.

## Author Contributions

RF-U: conceptualization, formal analysis, methodology, writing—original draft, and writing—review and editing. PM-C and GS-P: methodology, data curation, data analysis, writing—original draft, and writing—review and editing. MC-H: formal analysis and writing—review and editing. All authors contributed to the article and approved the submitted version.

## Conflict of Interest

The authors declare that the research was conducted in the absence of any commercial or financial relationships that could be construed as a potential conflict of interest.

## Publisher’s Note

All claims expressed in this article are solely those of the authors and do not necessarily represent those of their affiliated organizations, or those of the publisher, the editors and the reviewers. Any product that may be evaluated in this article, or claim that may be made by its manufacturer, is not guaranteed or endorsed by the publisher.

## References

[B1] Abad F. Olea J. Ponsoda V. García C. (2011). *Medición en Ciencias Sociales y de la Salud.* Madrid: Síntesis.

[B2] Ajayi A. Ismail K. Akpan W. (2019). Factors associated with consistent condom use: a cross-sectional survey of two Nigerian universities. *BMC Public Health* 19:1207. 10.1186/s12889-019-7543-1 31477068 PMC6719351

[B3] Ajzen I. Madden T. J. (1986). Prediction of goal-directed behavior: attitudes, intentions, and perceived behavioral control. *J. Exp. Soc. Psychol.* 22 453–474.

[B4] American Educational Research Association, American Psychological Association, and National Council on Measurement in Education (2014). *Standards for Educational and Psychological Testing.* Washington, DC: American Educational Research Association.

[B5] Asparouhouv T. Muthén B. (2009). Exploratory structural equation modeling. *Struct. Equ. Modeling* 16 397–438. 10.1080/10705510903008204

[B6] Ato M. López-García J. Benavente A. (2013). Un sistema de clasificación de los diseños de investigación en psicología. *Anal. Psicol.* 29 1038–1059. 10.6018/analesps.29.3.178511

[B7] Barendse M. Oort F. Timmerman M. (2015). Using exploratory factor analysis to determine the dimensionality of discrete responses. *Struct. Equ. Modeling* 22 87–101. 10.1080/10705511.2014.934850

[B8] Barrera-Herrera A. Vinet E. (2017). Adultez emergente y características culturales de la etapa en universitarios chilenos. *Ter. Psicol*. 35 47–56. 10.4067/S0718-48082017000100005 27315006

[B9] Beachy S. Lechuga J. Dickson-Gomez J. Liang C. (2020). Validation of brief condom use attitudes scales for spanish speaking people-who-use-drugs in El Salvador. *Research Square* [Preprint] 10.21203/rs.3.rs-20069/v1 35508750

[B10] Brown I. (1984). Development of a scale to measure attitude toward the condom as a method of birth control. *J. Sex Res.* 20 255–263. 10.1080/00224498409551224

[B11] Bryan A. Norris J. Abdallah D. Zawacki T. Morrison D. George W. (2017). Condom-insistence conflict in Women’s alcohol-involved sexual encounters with a new male partner. *Psychol. Women Q.* 41 100–113. 10.1177/0361684316668301 29720782 PMC5927388

[B12] Cáceres K. Pino R. (2018). Estimaciones poblacionales sobre VIH en Chile 2017 SPECTRUM ONUSIDA. *Rev. Chil. Infectol.* 35 642–648. 10.4067/S071610182018000600642 31095184

[B13] Cáceres-Burton K. (2019). Informe: situación epidemiológica de las infecciones de transmisión sexual en Chile, 2017. *Rev. Chil. Infectol*. 36 221–233. 10.4067/S0716-10182019000200221 31344158

[B14] Casique I. (2019). Gender differences in the sexual well-being of Mexican adolescents. *Int. J. Sex Health* 31 1–16. 10.1080/19317611.2018.1561587

[B15] Chen F. (2007). Sensitivity of goodness of fit indexes to lack of measurement invariance. *Struct. Equ. Modeling* 14 464–504. 10.1080/10705510701301834

[B16] Cho E. Kim S. (2015). Cronbach’s coefficient alpha: well known but poorly understood. *Organ. Res. Methods* 18 207–230. 10.1177/1094428114555994

[B17] Cohen J. (1988). *Statistical Power Analysis for the Behavioural Sciences*, 2nd Edn. New York, NY: Routledge.

[B18] Crosby R. Graham C. Milhausen R. Sanders S. Yarber W. (2010). “Condom use errors/problems survey,” in *Handbook of Sexuality-Related Measures*, eds Fisher T. D. Davis C. Yarber W. Davis S. (New York, NY: Routledge), 153–159. 10.1097/OLQ.0000000000000356

[B19] Davis K. C. Kirwan M. Wegner R. Neilson E. C. Stappenbeck C. A. (2020). Effects of alcohol, condom request style, and state anger on men’s condom use resistance. *J. Stud. Alcohol Drugs* 81 454–461. 10.15288/jsad.2020.81.454 32800081 PMC7437556

[B20] De Torres R. (2020). Facilitators and barriers to condom use among Filipinos: a systematic review of literature. *Health Promot. Perspect.* 10:306. 10.34172/hpp.2020.49 33312926 PMC7722996

[B21] DiStefano C. Morgan G. (2014). A comparison of diagonal weighted least squares robust estimation techniques for ordinal data. *Struct. Equ. Modeling* 21 425–438. 10.1080/10705511.2014.915373

[B22] Elshiekh H. Hoving C. de Vries H. (2020). Exploring determinants of condom use among university students in Sudan. *Arch. Sex. Behav.* 49 1379–1391. 10.1007/s10508-019-01564-2 32056040 PMC7145779

[B23] Ferrer-Urbina R. Leal-Soto F. Bravo N. Huaranca C. Perez J. Salinas T. (2018). Scale of risk behaviors, associated with STI / HIV-AIDS, for young Chileans. *Eur. Proc. Soc. Behav. Sci.* 60 800–809. 10.15405/epsbs.2019.04.02.99

[B24] Fishbein M. Ajzen I. (1975). *Belief, Attitude, Intention, and Behavior: An Introduction to Theory and Research.* Reading, MA: Addison-Wesley.

[B25] Gaborieau J. B. Pronello C. (2021). Validation of a unidimensional and probabilistic measurement scale for pro-environmental behaviour by travellers. *Transportation* 48 555–593. 10.1007/s11116-019-10068-w

[B26] Habel M. Leichliter J. Dittus P. Spicknall I. Aral S. (2018). Heterosexual anal and oral sex in adolescents and adults in the United States, 2011–2015. *Sex. Transm. Dis.* 45:775. 10.1097/OLQ.0000000000000889 29965947 PMC6753934

[B27] Hanna K. (1999). An adolescent and young adult condom self-efficacy scale. *J. Pediatr. Nurs.* 14 59–66. 10.1016/S0882-5963(99)80061-X 10063250

[B28] Helweg-Larsen M. Collins B. (1994). The UCLA multidimensional condom attitudes scale: documenting the complex determinants of condom use in college students. *Health Psychol.* 13 224–237. 10.1037/0278-6133.13.3.224 8055858

[B29] Hollub A. Reece M. Herbenick D. Hensel D. Middlestadt S. (2011). College students and condom attitude: validation of the multi-factor attitude toward condoms scale (MFACS). *J. Am. Coll. Health* 59 708–714. 10.1080/07448481.2010.546462 21950251

[B30] Instituto de Salud Pública (2016). *Boletín Vigilancia de Laboratorio. Resultados Confirmación de Infecciones por VIH en Chile, 2010–2015.* Available online at: https://www.ispch.cl/sites/default/files/BoletinVIH-15112017A.pdf (accessed May 04, 2021)

[B31] Instituto Nacional de Estadística (2018). *Síntesis de Resultados CENSO 2017.* Available online at: https://www.censo2017.cl/descargas/home/sintesis-de-resultados-censo2017.pdf (accessed May 04, 2021)

[B32] Janulis P. Newcomb M. Sullivan P. Mustanski B. (2017). Evaluating HIV knowledge questionnaires among men who have sex with men: a multi-study item response theory analysis. *Arch. Sex. Behav.* 47 107–119. 10.1007/s10508-016-0910-4 28488126 PMC5680146

[B33] Javier S. Abrams J. Moore M. Belgrave F. (2018). Condom use efficacy and sexual communication skills among African American college women. *Health Promot. Pract.* 19 287–294. 10.1177/1524839916676253 29451031

[B34] Johnson W. O’Leary A. Flores S. (2018). Per-partner condom effectiveness against HIV for men who have sex with men. *AIDS* 32 1499–1505. 10.1097/QAD.0000000000001832 29794493

[B35] Joint United Nations Program on HIV/AIDS (2019). *UNAIDS Data 2019.* Available online at: https://www.unaids.org/en/resources/documents/2019/2019-UNAIDS-data (accessed May 04, 2021)

[B36] Kilwein T. Looby A. (2018). Predicting risky sexual behaviors among college student drinkers as a function of event-level drinking motives and alcohol use. *Addict. Behav*. 76 100–105. 10.1016/j.addbeh.2017.07.032 28777973

[B37] Kilwein T. Kern S. Looby A. (2017). Interventions for alcohol-related risky sexual behaviors among college students: a systematic review. *Psychol. Addict. Behav.* 31 944–950. 10.1037/adb0000294 28639814

[B38] Kim Y. Min H. Lee J. Kim S. (2021). Una revisión integradora de estudios sobre el uso de condones entre estudiantes universitarios coreanos. *Invest. Enferm. Salud Infantil*. 27 43–55. 10.4094/chnr.2021.27.1.43

[B39] La Trobe H. L. Acott T. G. (2000). A modified NEP/DSP environmental attitudes scale. *J. Environ. Educ.* 32 12–20. 10.1080/00958960009598667

[B40] Li C. (2016). Confirmatory factor analysis with ordinal data: comparing robust maximum likelihood and diagonally weighted least squares. *Behav. Res. Methods* 48 936–949. 10.3758/s13428-015-0619-7 26174714

[B41] Mbelle N. Mabaso M. Chauke T. Sigida S. Naidoo D. Sifunda S. (2018). Perception and attitudes about male and female condom use amongst university and technical and vocational education and training (TVET) college students in South Africa: a qualitative enquiry of the 2014 higher education and training HIV/AIDS (HEAIDS) programme first things first campaign. *J. HIV AIDS* 4 1–8.

[B42] Mileti F. Mellini L. Sulstarova B. Villani M. Singy P. (2018). Exploring barriers to consistent condom use among sub-Saharan African young immigrants in Switzerland. *AIDS Care* 31 113–116. 10.1080/09540121.2018.1526371 30244601

[B43] Ministerio de Salud (2017). *ENCUESTA NACIONAL DE SALUD 2016-2017, Primeros Resultados.* Available online at: https://www.minsal.cl/wp-content/uploads/2017/11/ENS-2016-17_PRIMEROS-RESULTSADOS.pdf (accessed May 04, 2021)

[B44] Muthén B. Asparouhov T. (2012). Bayesian structural equation modeling: a more flexible representation of substantive theory. *Psychol. Methods* 17:313. 10.1037/a0026802 22962886

[B45] Muthén L. Muthén B. (1998–2017). *Mplus User’s Guide*, 8th Edn. Los Angeles, CA: Muthén & Muthén.

[B46] Neilands T. Choi K. (2002). A validation and reduced form of the female condom attitudes scale. *AIDS Educ. Prev.* 14 158–171. 10.1521/aeap.14.2.158.23903 12000233

[B47] Otzen T. Manterola C. (2017). Técnicas de Muestreo sobre una Población a Estudio. *Int. J. Morphol.* 35 227–232. 10.4067/S0717-95022017000100037 27315006

[B48] Pan American Health Organization (2019). *Situación de la Epidemia de la Infección por el VIH y Respuesta, América Latina y el Caribe, 2019.* Available online at: https://www.paho.org/hq/index.php?option=com_docman&view=download&category_slug=datos-estadisticos-5691&alias=51070-situacion-de-la-epidemia-de-la-infeccion-por-el-vih-y-respuesta-america-latina-y-el-caribe-2019&Itemid=270&lang=es (accessed May 04, 2021)

[B49] Patterson Y. (2019). Intragroup differences among African jamaican and African American women: empowerment, male condom-use intentions and negotiation. *Soc. Work Public Health* 34 1–19. 10.1080/19371918.2019.1589612 30916619

[B50] Pinyaphong J. Srithanaviboonchai K. Chariyalertsak S. Phornphibul P. Tangmunkongvorakul A. Musumari P. (2018). Inconsistent condom use among male university students in northern Thailand. *Asia Pac. J. Public Health* 30 147–157. 10.1177/1010539517753931 29409333

[B51] Plaza-Vidal R. Ibagon-Parra M. Vallejo-Medina P. (2020). Spanish translation, adaptation, and validation of the multidimensional condom attitudes scale with young Colombian men and women. *Arch. Sex. Behav.* 50 2729–2740. 10.1007/s10508-020-01759-y 32588255

[B52] Sacco W. Levine B. Reed D. Thompson K. (1991). Attitudes about condom use as an AIDS-relevant behavior: their factor structure and relation to condom use. *J. Consult. Clin. Psychol.* 3 265–272. 10.1037/1040-3590.3.2.265

[B53] Schreiber J. B. (2017). Update to core reporting practices in structural equation modeling. *Res. Soc. Adm. Pharm.* 13 634–643. 10.1016/j.sapharm.2016.06.006 27567146

[B54] Shamu S. Khupakonke S. Farirai T. Slabbert J. Chidarikire T. Guloba G. (2020). Knowledge, attitudes and practices of young adults towards HIV prevention: an analysis of baseline data from a community-based HIV prevention intervention study in two high HIV burden districts, South Africa. *BMC Public Health* 20:1249. 10.1186/s12889-020-09356-3 32807116 PMC7433171

[B55] Sheeran P. Abraham C. Orbell S. (1999). Psychosocial correlates of heterosexual condom use: a meta-analysis. *Psychol. Bull.* 125 90–132. 10.1037/0033-2909.125.1.90 9990846

[B56] Sheeran P. Maki A. Montanaro E. Avishai-Yitshak A. Bryan A. Klein W. M. (2016). The impact of changing attitudes, norms, and self-efficacy on health-related intentions and behavior: a meta-analysis. *Health Psychol.* 35 1178–1188. 10.1037/hea0000387 27280365

[B57] Smith D. Herbst J. Zhang X. Rose C. E. (2015). Condom effectiveness for HIV prevention by consistency of use among men who have sex with men in the United States. *J. Acquir. Immune Defic. Syndr.* 68 337–344. 10.1097/QAI.0000000000000461 25469526

[B58] Teye-Kwadjo E. Kagee A. Swart H. (2017). Predicting the intention to use condoms and actual condom use behaviour: a three-wave longitudinal study in Ghana. *Appl. Psychol. Health Well Being* 9 81–105. 10.1111/aphw.12082 27925435 PMC5659181

[B59] The Jamovi Project (2020). *Jamovi (Version 1.8.1) [Computer Software].* Available online at: https://www.jamovi.org (accessed 26 March 2021)

[B60] Vallejo-Medina P. Ramírez E. Saavedra-Roa A. Gómez-Lugo M. Pérez-Durán C. (2019). Spanish validation of female condom attitude scale and female condom use in Colombian young women. *BMC Women’s Health* 19:128. 10.1186/s12905-019-0825-z 31660933 PMC6819378

[B61] Viladrich C. Angulo-Brunet A. Doval E. (2017). A journey around alpha and omega to estimate internal consistency reliability. *Anal. Psicol*. 33 755–782. 10.6018/analesps.33.3.268401

[B62] World Health Organization (2016). *Proyecto de Estrategia Mundial del Sector de la Salud Contra las Infecciones de Transmisión Sexual Para 2016-2021.* Available online at: http://www.who.int/reproductivehealth/GHSS_STI_SP_06012016.pdf (accessed May 04, 2021)

[B63] Xiao Y. Liu H. Hau K. (2019). A comparison of CFA, ESEM, and BSEM in test structure analysis. *Struct. Equ. Modeling* 26 665–677. 10.1080/10705511.2018.1562928

[B64] Yi S. Te V. Pengpid S. Peltzer K. (2018). Social and behavioural factors associated with risky sexual behaviours among university students in nine ASEAN countries: a multi-country cross-sectional study. *SAHARA J.* 15 71–79. 10.1080/17290376.2018.1503967 30058474 PMC6070966

[B65] Zanna M. P. Rempel J. K. (1988). “Attitudes: a new look at an old concept,” in *The Social Psychology of Knowledge*, eds Bar-Tal D. Kruglanski A. W. (Cambridge: Cambridge University Press), 315–334.

