# Supplementary material for: Development and Evidence of the Validity of the Condom Use Attitudes Scale for Youth and Adults in a Chilean Context
Source: Front Psychol. 2021 Dec 1;12:727499. doi: 10.3389/fpsyg.2021.727499 (PMC8671144; doi:10.3389/fpsyg.2021.727499)
Supplement: Supplementary file 1 [file Table_1.docx]

**SUPPLEMENTARY MATERIAL**

|  |  | Students | General population |
| --- | --- | --- | --- |
|  |  | *M* (*SD*) o n (%) | *M* (*SD*) o n (%) |
| Biological sex | Male | 253 (48.6%) | 464 (46.7%) |
|  | Female | 264 (50.7%) | 514 (52.0%) |
|  | Missing | 3 (0.7%) | 12 (1.3%) |
| Age (years) |  | 22.78 (3.56) | 23.33 (4.68) |
| Marital status | Single | 484 (93.0%) | 873 (88,0%) |
|  | Married | 17 (3.2%) | 74 (7.4%) |
|  | Stable couple | 17 (3.2%) | 28 (2.8%) |
|  | Missing | 2 (0.6%) | 17 (1.8%) |
| Sexual orientation | Heterosexual | 438 (84.2%) | 818 (82.4%) |
|  | Homosexual | 33 (6.3%) | 28 (2.8%) |
|  | Bisexual | 30 (5.7%) | 34 (3.4%) |
|  | Other | --- | 2 (0.2%) |
|  | Missing | 19 (3.8%) | 110 (11.2%) |
| Number of sexual partners |  | 5.34 (8.01) | 6.27 (10.71) |
| Diagnosed with HIV/AIDS | Yes | 2 (0.4%) | 6 (0.6%) |
|  | No | 516 (99.2%) | 974 (98.2%) |
|  | Missing | 2 (0.4%) | 12 (1,2%) |
| In the last 2 years, they have used protective barrier methods | Yes, regularly | 320 (61.3%) | 565 (56.8%) |
|  | Never | 191 (36.5%) | 377 (37.9%) |
|  | Missing | 9 (2.2%) | 50 (5.3%) |
| HIV/AIDS test performed | Yes, regularly | 248 (48.2%) | 447 (45.0%) |
|  | Never | 265 (50.8%) | 527 (53.0%) |
|  | Missing | 7 (1.0%) | 18 (2.0%) |
| HIV/AIDS test requested from your sexual partner | Yes, regularly | 186 (35.5%) | 304 (30.5%) |
|  | Never | 324 (62.4%) | 659 (66.6%) |
|  | Missing | 10 (2.1%) | 29 (2.9%) |
| Diagnosed with STIs | Never | 493 (94.8%) | 934 (94.1%) |
|  | Only once | 19 (3.6%) | 37 (3.7%) |
|  | Twice | 1 (0.2%) | 5 (0.5%) |
|  | Missing | 7 (1.4%) | 16 (1.7%) |

**Table S1.** Socio-demographic characteristics of the study simples.

*M* = Mean; *SD* = Standard deviation; n = Number of subjects; % = Percentage.


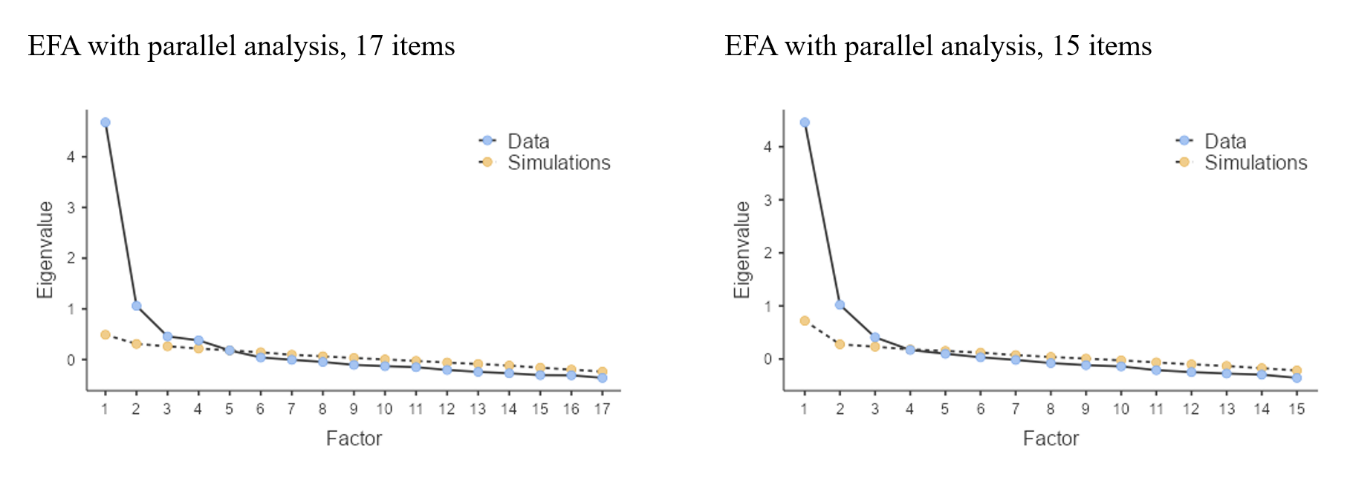


**Figure S2.** Scree plot of the EFA with parallel analysis, sample (a).

| Attitude towards condoms | Factor Loadings EFA | | | Factor Loadings ESEM^1^ | | |
| --- | --- | --- | --- | --- | --- | --- |
|  | A | B | C | A | B | C |
| Affective (A) |  |  |  |  |  |  |
| Es difícil disfrutar del sexo cuando se usa preservativo | **.852** | -.012 | -.012 | **.917** | -.066 | .015 |
| Siento que el preservativo disminuye mi satisfacción sexual. | **.863** | .044 | -.021 | **.945** | -.004 | -.021 |
| Los preservativos quitan el romanticismo. | **.276** | .208 | .219 | **.635** | .021 | .130 |
| Las personas obtienen más placer en las relaciones sexuales sin preservativo. | **.628** | .041 | .065 | **.353** | .164 | .263 |
| Behavioral (B) |  |  |  |  |  |  |
| Evito usar preservativo cada vez que me lo permiten. | .167 | **.617** | -.022 | .379 | **.576** | -.010 |
| No suelo llevar preservativos cuando tengo un encuentro sexual. | -.007 | **.615** | .063 | .220 | **.510** | .104 |
| Uso preservativo solo con las personas que me dan desconfianza. | .038 | **.290** | .303 | .027 | **.272** | .433 |
| Tendría relaciones sexuales aun cuando mi pareja se negará a usar preservativo. | .136 | **.426** | .119 | .239 | **.372** | .179 |
| Cognitive (C) |  |  |  |  |  |  |
| Creo que el preservativo debieran usarlo solo las personas promiscuas. | -.086 | -.001 | **.626** | -.310 | -.015 | **.892** |
| El uso de preservativos es solo para relaciones pasajeras. | .019 | -.008 | **.694** | -.182 | -.005 | **.891** |
| Pienso que el preservativo es innecesario en las personas sanas. | -.048 | .088 | **.746** | -.238 | .077 | **.940** |
| Pienso que el uso del preservativo no es necesario en una relación estable. | .054 | .318 | **.399** | -.005 | .247 | **.582** |
| Creo que sugerir el uso del preservativo genera desconfianza. | .104 | .053 | **.443** | .014 | .025 | **.616** |
| Considero que el preservativo no es un método confiable para la prevención del VIH/SIDA. | .179 | -.255 | **.429** | .012 | -.308 | **.592** |
| Los preservativos tienen poca utilidad. | .204 | -.134 | **.469** | .091 | -.197 | **.634** |
|  | Correlations | | | Correlations | | |
| Affective (A) | – |  |  | – |  |  |
| Behavioral (B) | .564** | – |  | **.372**** | – |  |
| Cognitive (C) | .391** | .440** | – | **.561**** | **.464**** | – |

**Table S3.** Factor Loadings of the CUEAS from EFA and ESEM with 15 items in university student’s sample.

^1^ = Factor loadings from ESEM with three covariate factors, 15 items solution (M1).

**Table S4.** Fit Indexes for ESEM, CFA and Multiple–group ESEM.

|  | Par | χ^2^ | *df* | χ^2^/ *df* | RMSEA | 90% CI | CFI | TLI | SRMR | CMs | Δ χ^2^ (Δ *_df_*) | *p* | Δ CFI | Δ RMSEA |
| --- | --- | --- | --- | --- | --- | --- | --- | --- | --- | --- | --- | --- | --- | --- |
|  |  |  |  |  |  |  |  |  |  |  |  |  |  |  |
| Single–group ESEM/CFA |  |  |  |  |  |  |  |  |  |  |  |  |  |  |
| M1 Three–factors ^a^ | 87 | 179.230 | 63 | 2.844 | .060 | [.049, .070] | .982 | .969 | .031 | – | – | – | – | – |
| M2a Three–factors revised ^a^ | 57 | 61.566 | 18 | 3.420 | .068 | [.050, .087] | .992 | .979 | .023 | – | – | – | – | – |
| M2b Three–factors revised ^b^ | 57 | 71.996 | 18 | 3.999 | .055 | [.042, .069] | .994 | .984 | .016 | – | – | – | – | – |
| M3 One-factor ^b^ | 40 | 1480.23 | 35 | 42.29 | .205 | [.196, .214] | .829 | .780 | .115 | – | – | – | – | – |
|  |  |  |  |  |  |  |  |  |  |  |  |  |  |  |
| Multi-group ESEM ^1^ |  |  |  |  |  |  |  |  |  |  |  |  |  |  |
| M4 Configural invariance | 94 | 77.559 | 36 | 2.154 | .049 | [.034, .064] | .987 | .967 | .019 | – | – | – | – | – |
| M5 Metric invariance | 73 | 123.732 | 57 | 2.170 | .049 | [.037, .061] | .979 | .966 | .035 | M4–M5 | 46.173 (21) | .001* | -.008 | .000 |
| M6 Scalar invariance | 66 | 154.652 | 64 | 2.416 | .054 | [.043, .065] | .971 | .959 | .038 | M4–M6 | 77.093 (28) | .000* | -.016 | .005 |

^a^ = Student sample (a); ^b^ = General population sample (b); M1 = ESEM with three covariate factors, 15 items; M2a = ESEM with three covariate factors, 10 items; M2b = ESEM with three covariate factors, 10 items; M3 = One-dimensional CFA, 10 items ^1^ = Invariance between boys’ and girls’ students; χ2 = Chi-square; *df* = Degree of freedom; RMSEA = Root Mean Square Error of Approximation; CI = 90% Confidence Interval; CFI = Comparative Fit Index; TLI = Tucker-Lewis Index; SRMR = Standardized Root Mean Square Residual; CMs = Comparisons between models; Δ χ^2^ = Change in chi-square; Δ *_df_* = Change in degrees of freedom; *p* = Significance; * = *p* < .001; Δ CFI = Change in comparative adjustment index; Δ RMSEA = Change in the error of the mean square of the approximation root.

| Attitude towards condoms | Mean (*SD*) | S | K | Factor Loadings^1^ | | |
| --- | --- | --- | --- | --- | --- | --- |
|  |  |  |  | A | B | C |
| Affective (A) |  |  |  |  |  |  |
| Es difícil disfrutar del sexo cuando se usa preservativo  (It's hard to enjoy sex when you use a condom). | 2.31 (0.90) | 1.64 | -3.48 | **.916**** | -.004 | .036 |
| Siento que el preservativo disminuye mi satisfacción sexual.  (I feel that the condom decreases my sexual satisfaction). | 2.37 (0.92) | 0.23 | -4.04 | **.827**** | .136 | -.010 |
| Las personas obtienen más placer en las relaciones sexuales sin preservativo.  (People get more pleasure from sex without a condom). | 2.51 (1.06) | -0.37 | -5.68 | **.555**** | .223* | .003 |
| Behavioral (B) |  |  |  |  |  |  |
| Evito usar preservativo cada vez que me lo permiten.  (I avoid using a condom every time I'm allowed). | 1.95 (0.98) | 6.11 | -3.27 | .006 | **.830**** | -.075 |
| No suelo llevar preservativos cuando tengo un encuentro sexual.  (I don't usually wear a condom when I have a sexual encounter). | 2.01 (1.02) | 5.32 | -4.11 | -.063 | **.712**** | .014 |
| Tendría relaciones sexuales aun cuando mi pareja se negará a usar preservativo.  (I would have sex even if my partner refused to use a condom). | 2.15 (1.03) | 3.64 | -4.83 | .040 | **.588**** | .046 |
| Cognitive (C) |  |  |  |  |  |  |
| Creo que el preservativo debieran usarlo solo las personas promiscuas.  (I think the condom should only be used by promiscuous people). | 1.45 (0.76) | 15.62 | 10.1 | -.078 | .017 | **.789**** |
| El uso de preservativos es solo para relaciones pasajeras.  (The use of condoms is only for temporary relations). | 1.61 (0.88) | 11.54 | 2.64 | .110 | -.045 | **.798**** |
| Pienso que el preservativo es innecesario en las personas sanas.  (I think condoms are unnecessary in healthy people). | 1.54 (0.80) | 13.37 | 6.57 | -.012 | .119 | **.791**** |
| Creo que sugerir el uso del preservativo genera desconfianza.  (I think that suggesting condom use creates distrust). | 1.59 (0.80) | 11.40 | 3.41 | .074 | .168 | **.472**** |
|  |  |  |  | Correlations | | |
| Affective (A) | 2.40 (0.83) | 0.39 | -3.39 | – |  |  |
| Behavioral (B) | 2.04 (0.78) | 4.40 | -1.80 | **.645**** | – |  |
| Cognitive (C) | 1.55 (0.60) | 10.28 | 4.25 | **.287**** | **.503**** | – |

**Table S5.** Descriptive Information of the CUAS and Factor Loadings Resulting from ESEM in university student’s sample.

*SD* = Standard Deviation; S = Skewness; K = Kurtosis; ** = *p* < .001; ^1^ = Factor loadings from ESEM with three covariate factors, 10 items solution (M2a).


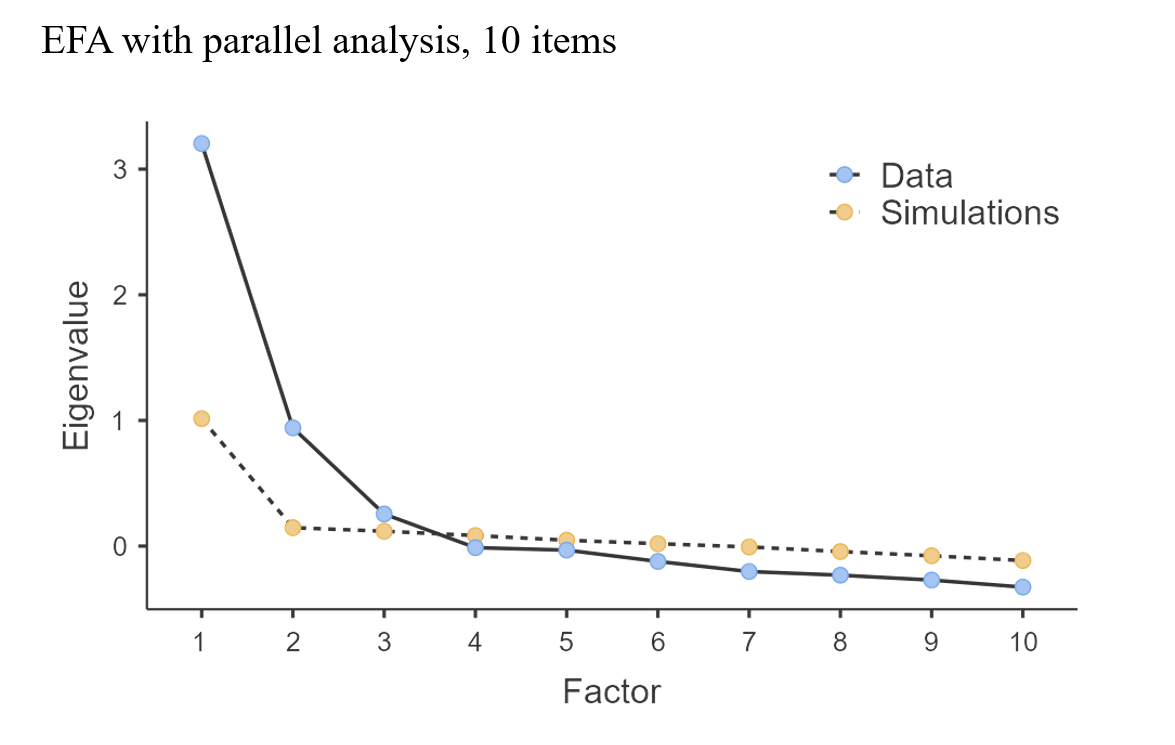


**Figure S6.** Scree plot of the EFA with parallel analysis, sample (b).

**Table S7.** Standardized effects of the latent dimensions of the CUES on the sexual risk behaviors scale and condom use.

|  | SAMP | IUBP | SAIAD | Condom Use |
| --- | --- | --- | --- | --- |
| Affective | **.216**** | .015 | **.237**** | **.141*** |
| Behavioral | .009 | **.736**** | .015 | **-.452**** |
| Cognitive | -.076 | **-.455**** | .023 | -.049 |

* = p < .05; ** = < .001; Samp = Sexual activity with multiple partners; Iubp = Inappropriate use of protective barriers; Saiad = Sexual activity under the influence of alcohol or drugs
