# Supplementary material for: Development and Evidence of the Validity of the Condom Use Attitudes Scale for Youth and Adults in a Chilean Context
Source: Front Psychol. 2021 Dec 1;12:727499. doi: 10.3389/fpsyg.2021.727499 (PMC8671144; doi:10.3389/fpsyg.2021.727499)
Supplement: Supplementary file 2 [file Table_2.DOCX]

**SUPPLEMENTARY MATERIAL**

**S1 Protocol: Spanish version scale**

Estimada(o):

A continuación, se presenta una serie de afirmaciones que pueden representar diferentes opiniones sobre el uso del preservativo. Por favor, señale con una “X”, en qué medida estas afirmaciones representan tú opinión, indicando: **TOTALMENTE EN DESACUERDO** si la opinión referida es muy distinta a la suya; **EN DESACUERDO** si su desacuerdo es leve; **DE ACUERDO** si lo representa ligeramente; y **TOTALMENTE DE ACUERDO** si lo representa totalmente.

| N.º | ÍTEMS | Totalmente en desacuerdo | En desacuerdo | De acuerdo | Totalmente de acuerdo |
| --- | --- | --- | --- | --- | --- |
| 1 | Es difícil disfrutar del sexo cuando se usa preservativo. |  |  |  |  |
| 2 | Siento que el preservativo disminuye mi satisfacción sexual. |  |  |  |  |
| 3 | Las personas obtienen más placer en las relaciones sexuales sin preservativo. |  |  |  |  |
| 4 | Evito usar preservativo cada vez que me lo permiten. |  |  |  |  |
| 5 | No suelo llevar preservativos cuando tengo un encuentro sexual. |  |  |  |  |
| 6 | Tendría relaciones sexuales aun cuando mi pareja se negará a usar preservativo. |  |  |  |  |
| 7 | Creo que el preservativo debieran usarlo solo las personas promiscuas. |  |  |  |  |
| 8 | El uso de preservativos es solo para relaciones pasajeras. |  |  |  |  |
| 9 | Pienso que el preservativo es innecesario en las personas sanas. |  |  |  |  |
| 10 | Creo que sugerir el uso del preservativo genera desconfianza. |  |  |  |  |

**S2 Protocol: unofficial English translated scale (translation for guidance only)**

Dear:

Below are a number of statements that may represent different opinions about condom use. Please mark with an "X" the degree to which these statements represent your opinion, indicating: **STRONGLY DISAGREE** if the opinion referred to is very different from yours; **DISAGREE** if your disagreement is slight; **AGREE** if it represents it slightly; and **STRONGLY AGREE** if it represents it completely.

| N.º | ITEMS | Strongly disagree | Disagree | Agree | Strongly agree |
| --- | --- | --- | --- | --- | --- |
| 1 | It's hard to enjoy sex when you use a condom. |  |  |  |  |
| 2 | I feel that the condom decreases my sexual satisfaction. |  |  |  |  |
| 3 | People get more pleasure from sex without a condom. |  |  |  |  |
| 4 | I avoid using a condom every time I'm allowed. |  |  |  |  |
| 5 | I don't usually wear a condom when I have a sexual encounter. |  |  |  |  |
| 6 | I would have sex even if my partner refused to use a condom. |  |  |  |  |
| 7 | I think the condom should only be used by promiscuous people. |  |  |  |  |
| 8 | The use of condoms is only for temporary relations. |  |  |  |  |
| 9 | I think condoms are unnecessary in healthy people. |  |  |  |  |
| 10 | I think that suggesting condom use creates distrust. |  |  |  |  |
